# Towards enhancing national capacity for evidence informed policy and practice in falls management: a role for a "Translation Task Group"?

**DOI:** 10.1186/1743-8462-4-6

**Published:** 2007-05-31

**Authors:** Roslyn G Poulos, Anthony B Zwi, Stephen R Lord

**Affiliations:** 1 The School of Public Health and Community Medicine, The University of New South Wales, Sydney, 2052, Australia; 2 The Prince of Wales Medical Research Institute, Barker St, Randwick, 2031, Australia

## Abstract

**Background:**

There has been a growing interest over recent years, both within Australia and overseas, in enhancing the translation of research into policy and practice. As one mechanism to improve the dissemination and uptake of falls research into policy and practice and to foster the development of policy-appropriate research, a "Falls Translation Task Group" was formed as part of an NHMRC Population Health Capacity Building grant. This paper reports on the group's first initiative to address issues around the research to policy and practice interface, and identifies a continuing role for such a group.

**Methods:**

A one day forum brought together falls researchers and decision-makers from across the nation to facilitate linkage and exchange. Observations of the day's proceedings were made by the authors. Participants were asked to complete a questionnaire at the commencement of the forum (to ascertain expectations) and at its completion (to evaluate the event). Observer notes and the questionnaire responses form the basis of analysis.

**Results:**

Both researchers and decision-makers have a desire to bridge the gap between research and policy and practice. Significant barriers to research uptake were highlighted and included both "health system barriers" (for example, a lack of financial and human resources) as well as "evidence barriers" (such as insufficient economic data and implementation research). Solutions to some of these barriers included the identification of clinical champions within the health sector to enhance evidence uptake, and the sourcing of alternative funding to support implementation research and encourage partnerships between researchers, decision-makers and other stakeholders.

**Conclusion:**

Participants sought opportunities for ongoing networking and collaboration. Two activities have been identified as priorities: establishing a "policy-sensitive" research agenda and partnering researchers and decision-makers in the process; and establishing a National Translation Task Group with a broad membership.

## Background

There has been increasing international interest over the past decade or so in the transfer and uptake of research into policy and practice [1]. Initially, this interest centered on clinical decision-making (evidence-based medicine), but more recently it has come to include health service managers and policymakers (evidence-based health service management, and evidence-based policy making) [1,2]. While the expectation is that the best available evidence will be used to inform decisions, the rhetoric continues to exceed the reality [3]. Some of the problem is attributed to the "cultural" differences between those who do research, and those who may be in a position to use it [3]. In a recent systematic review, personal contact between researchers and decision-makers, and the timeliness and relevance of research were the most frequently identified factors facilitating the uptake of research knowledge [4].

A National Health and Medical Research Council (NHMRC) review of injury as a national health priority area noted that "*missing pieces of evidence, poor transmission of research findings to those responsible for implementation, and limited skills of administrators and policymakers for interpreting research all contributed to the failure of research to inform practice*" in Australia [5]. Consequently, part of an NHMRC Capacity Building Grant in Injury, Trauma and Rehabilitation (2005–2009) awarded to a consortium of research institutions, is being directed towards improving the translation of injury research into policy and practice. As one mechanism to do this, we have formed *Translation Task Groups (TTGs) *across a number of injury areas, including falls in older people [6]. The growing public health impact of fall injury, a strong research base identifying effective interventions yet to be widely implemented, and a current level of commitment to falls prevention within the health sector, identified this as a priority area for action.

The Falls TTG aims to enhance linkage and exchange [3] between falls researchers, policymakers and other stakeholders, in order to foster the development of policy-appropriate research and to improve the dissemination and uptake of falls research into policy and practice. The membership of the Falls TTG currently comprises eight researchers from within our research consortium. Six members are falls researchers, and two (RP and AZ) are public health practitioners with research interests in the interface between research and policy and practice. At the first meeting of the Falls TTG, the group recognized the need for deliberate, planned bridging activities to facilitate communication and understanding between falls researchers and decision-makers [7], as a first step towards enhancing national capacity for evidence informed policy and practice in falls management. This conviction created the impetus for the forum described in this report.

A national meeting of policymakers and managers from state, territory and Australian Government Departments of Health was planned for early 2006, to consider implementation of the "National Falls Prevention for Older People Plan" [8]. Released in July 2005, the National Plan was developed in response to a predicted epidemic of falls and fall injury resulting from the projected demographic ageing of the population (Table 1). The Falls TTG hosted a one day forum on "translating falls research into policy and practice" alongside this meeting.

**Table 1:** Projected implications of fall related injury [21].

Over the next 50 years there will be a considerable increase in the proportion of the Australian population aged over 65 years of age. Unless effective preventive strategies are put in place, this will result in increased demands for health services for fall related injuries. By the year 2051 [21]:
▪ The projected costs of fall related injury in Australia will increase by almost three fold to $1375 million per annum.
▪ The equivalent of 2500 additional beds will be required for fall related injury treatment *and*
▪ An additional 3320 nursing home places will be needed.

This paper reports on the value of this opportunity to bring researchers, policymakers and managers together to consider issues around the research to policy and practice interface. It presents the issues identified and the outcomes achieved, and suggests a continuing role for a Falls Translation Task Group at a national level.

## Methods

The two day national meeting was held in Sydney in March, 2006. The first day was devoted to the joint meeting of researchers, policymakers and managers (Day One) and was the initiative of the Falls TTG. Those invited included a number of eminent Australian falls researchers, and policymakers and managers with responsibility for fall injury prevention from all the jurisdictions (hereafter called decision-makers). Several clinicians attended either in their roles as researchers or managers. The second meeting day was attended by a smaller group of decision-makers and dealt with the implementation of the National Plan. Day Two is outside the scope of this report.

The meeting was jointly planned and chaired by a researcher (SL) from the Falls TTG and a policymaker from the local jurisdiction. The objectives of the day were to:

1. document evidence of good practice in falls prevention and management

2. identify gaps in knowledge

3. discuss the barriers to, and solutions for, translating research into policy and practice, and

4. explicate ways in which researchers can support decision-makers.

Brief presentations by invited researchers covered findings related to falls risk factors and intervention strategies, and included discussions on the strength of evidence associated with each, and areas where research is lacking. One presentation dealt with interventions that have been proven to be ineffective. At the conclusion of each presentation, questions were taken from the floor. This was followed by a period of discussion between researchers and decision-makers on the barriers to implementing research into policy and practice, and drew upon the information gained from the research presentations. The final session of the day was dedicated to identifying strategies to overcome the implementation barriers. All participants were asked to complete a short questionnaire at the start of the day (to ascertain expectations), and at its completion (to evaluate the event).

Two members of the Falls TTG (RP and AZ) observed and noted the process, presentations and interactions between researchers and decision-makers. This paper presents these observations and an analysis of the participant questionnaires.

## Results

Twenty-seven persons attended, comprising 15 researchers and 12 decision-makers. This group included the majority of senior falls researchers in Australia, and decision-makers from the Australian Government and all but two of the State and Territory Health Departments.

### Translation and exchange

Researchers gave short and succinct presentations that distilled large amounts of information. Presentations were generally clear, although there was a tendency to use scientific concepts and technical terms (such as "forest plots" and "relative risks"), and to present complicated methodologies (for example, "factorial design studies") with minimal explanation. Description of the cost or feasibility of proposed interventions was not well covered. While final conclusions were drawn for each presentation, these were often qualified, making the policy implications uncertain. For example, the effectiveness of an intervention was qualified in terms of the category of allied health professional required to apply it, and/or the specific setting in which it was utilized; presenters were unwilling to venture suggestions about the applicability of research findings to sub-optimal conditions or to different contexts.

**Figure 1:**
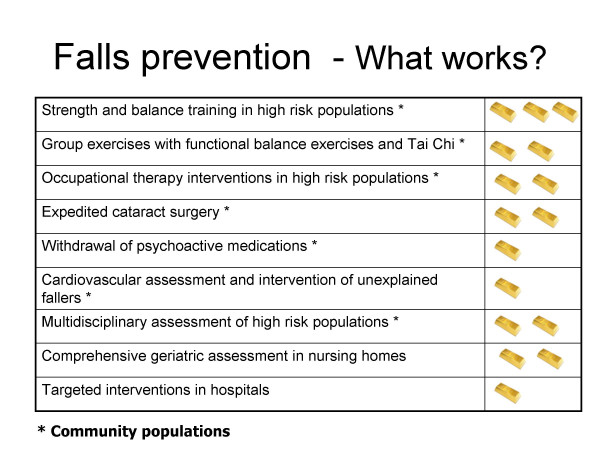
**Fall prevention interventions by "gold bar awards"**. Level and quality of evidence is represented by the number of gold bars, with three gold bars representing those interventions supported by the best evidence.

In a final presentation, an overall summary of all the research presented was given, using a simple system of "gold bar awards" (Figure 1). Bars were awarded on the basis of the researcher's opinion of the level and quality of evidence in support of an intervention, with three bars representing interventions supported by the best evidence. This visual summary appealed to participants and the concept of "gold bar interventions" was adopted in subsequent discussions between researchers and decision-makers.

The opportunity for dialogue with researchers was readily taken up by decision-makers. Questions to researchers had a very practical focus and concerned the ability to generalize results to different populations outside specific research settings, the size of reduction in risk which is clinically important, and the cost of interventions: all very relevant if application of research from one setting to another is to be achieved.

### Barriers to getting research into policy and practice

Researchers identified a number of barriers to translating falls research into policy and practice. These included: a failure of "people" (by inference, decision-makers and practitioners) to have in place systems to keep abreast of emerging research results or to read the key journals; the cost and feasibility of implementation; and the financial constraint imposed on researchers because funding bodies provide limited support, if any, for activities to enhance the translation of research into policy and practice.

Researchers perceived that both universities and funding bodies did not encourage "translation", putting great weight on scientific journal publication as the sole form of valued dissemination. A "silo" mentality among the research community was also identified, with researchers in falls prevention working in isolation from researchers in physical activity despite clear synergies between these fields.

The barriers identified by decision-makers included both system issues and evidence barriers. A lack of financial and human resources in the public health system for falls prevention, non-recurrent program funding, health system restructuring, and the individual "silo" mentality in health where synergies between chronic disease prevention programs were not exploited, were identified as important. It was also felt that a culture of pessimism among health care workers with regard to falls prevention in older people mitigates the uptake of evidence-based interventions within the health sector.

While the system issues were largely outside the control of the researchers, evidence barriers were not. Decision-makers indicated that they were limited in their ability to implement research evidence because of a failure by researchers to include economic evaluations alongside clinical evidence of efficacy. Decision-makers suggested that this gap undermined their ability to present the potentially "saleable" message of future financial savings thus reducing policy support and potential funding. Further, the lack of economic evaluation restricted the decision-making process since it was not possible to make comparisons of returns from alternative forms of investment. In discussion, the value of even "rough and ready" estimates of costs and of potential savings, in the absence of detailed studies, was promoted. The second identified barrier concerned a lack of "implementation research" to evaluate the methods used in promoting and implementing interventions in the field. Without this research, decision-makers felt they could not identify, institute or disseminate effective implementation strategies, thereby limiting the outcomes achieved and the return on investment.

### Solutions for getting research into policy and practice

Solutions to health system issues were not discussed in detail. However, the need to identify clinical champions was raised by decision-makers: such motivated individuals, leaders within their field, can encourage the uptake of research in practice. It was suggested that adequate representation from these champions characterize future meetings. Researchers agreed that basic economic data could be provided alongside indicators of clinical efficacy. More complex economic evaluations would require additional research funding and the involvement of health economists. Grants which encouraged partnerships between researchers, decision-makers and other stakeholders (such as ARC Linkage Grants or the NHMRC equivalents under consideration) were seen as a potential mechanism by which implementation research could be fostered.

Participant evaluations indicated that both groups sought to bridge the gap between research and policy and practice, and desired opportunities for ongoing networking and collaboration. The most frequently identified gain was that of new insights into the role of the other community. Suggestions on improving future meetings included widening the list of invited participants, and developing regular meetings. All responses are summarized in Table 2.

**Table 2:** Participant evaluations – pre and post-forum. (n = 10 DM; n = 6 R; authors excluded)

Pre-forum	**What were you hoping this day would achieve?**	
	
	Networking & collaboration	R(3) DM(5)
	Understanding & bridging the gap between research & policy & practice	R(3) DM(3)
	Evidence update	R(1) DM(6)
	Understanding the barriers and issues around implementation	DM(3)
	Dedicated staff to apply research	R(1)
	Understanding policymakers	R(2)
	
	**What do you consider the top 3 issues that need to be addressed today, in this meeting of researchers and policy makers?**	
	
	Input into the research agenda	DM(1)
	Translation of research into policy and practice	R(2) DM(3)
	Limitations to implementation	R(2) DM(2)
	Collaboration and partnerships	R(5) DM(4)
	Update evidence/information sharing	R(1) DM(4)
	Sustainability/funding	R(1) DM(4)
	Knowledge gaps	R(2) DM(1)
	Identify what policymakers need from researchers	R(1)
Post-forum	**Have you found this day worthwhile?**	
	
	Very worthwhile	R(2) DM(6)
	Somewhat worthwhile	R(3) DM(4)
	Not worthwhile	R(1)
	
	**What have you learned or gained from your participation today?**	
	
	Barriers and frustrations exist across all sectors	DM(1)
	Insights into policy and research processes	R(5) DM(3)
	Knowing what other jurisdictions are doing	DM(2)
	Evidence update	R(1) DM(2)
	Opportunity to network with researchers	DM(2)
	Need to improve communication between groups	R(1) DM(1)
	
	**How could a future meeting which brings researchers and policy makers together be improved?**	
	
	More clinician involvement	R(1)DM(3)
	Broader range of invitees	DM(2)
	More regular event	R(1) DM(1)
	More time	DM(2)
	Come with a specific list of problems that need solutions	R(2) DM(1)

R, researcher; DM, decision-maker; count (n)

## Discussion

Enhancing the interface between research and policy and practice is widely accepted as being crucial to the adoption and implementation of effective interventions [9]. As a first and modest step, enhancing opportunities for ongoing communication between researchers and policymakers are needed [10]. Bringing participants with different perspectives together in a local or national forum to exchange knowledge, create networks, and to identify important gaps in research evidence is clearly valuable. While not unique, such events are a necessary first step to building trust between different stakeholders.

The forum was successful in providing a distillation of evidence for decision-makers, and provided the opportunity for a discussion of research findings. There is evidence to suggest that providing users with an opportunity to discuss the implications of research with researchers, increases uptake [11]. However, researchers should pay careful attention to the needs of decision-makers both in terms of the content of the information, and in its presentation. Technical jargon should be minimized [7] and presentations which integrate results and are given in visually compelling and readily understandable formats (such as the "gold bar awards") should be encouraged [7,12]. While researchers tend to naturally focus on the complexity of issues and qualify conclusions [13], decision-makers need the policy implications of research to be clearly articulated [7]. Supporting economic data is also important to decision-making [12,14]. Developing a shared understanding of the particular context in which interventions might be considered, with health system, human and financial resource limitations and opportunity cost considerations in mind may help identify what can, or can not, be done in a given setting. Anticipating impediments, and jointly seeking to resolve them, provides a fruitful avenue for joint consideration and action.

The challenges and barriers are well recognized [15,16], but these have not previously been discussed in a combined forum of Australian falls researchers and decision-makers. Articulating them in this setting allowed researchers and decision-makers to appreciate the difficulties faced by their counterparts. For example, while they could do little to address the system barriers faced by decision-makers, it was enlightening for researchers to understand the complexities and constraints, the limited time availability, the high pressure and the modest resources in the health-care system, and the difficulties of implementing change on the ground [17]. Specifically, this led to an enhanced appreciation by researchers of the importance of economic evaluations and implementation research, and a commitment to act on these issues.

The failure of funding bodies to recognize the 'overhead' costs of linkage and exchange has been noted elsewhere as a significant limitation, and expanding the definition of research to include these activities has been suggested [18]. The process of initial consultation between researchers and decision-makers to inform the research question; ongoing linkages throughout the project to promote interest and maintain research relevance; and post-project dialogue and exchange between researchers and decision-makers should all be 'fundable' stages of the research process [18]. Fund-granting bodies should seek to make available funds for facilitating communication, exchange and collaboration across stakeholder organisations, and for ensuring that research findings can be communicated more effectively in plain language [19].

The TTG formalized the relationship between researchers with a common interest in facilitating the movement of research into policy and practice, and created an identifiable entity with the clear task of "translation". There is also an opportunity to build sustainable approaches. The long term nature of the NHMRC Capacity Building Grant (5 years) and the funding of dedicated person-time for translation of research into policy and practice enables further development and evaluation of processes to promote evidence informed policy and practice. A key limiting factor will be the number of, and resources for, formal opportunities for linkage and exchange at a national level. The first forum was linked to an already planned national meeting of policymakers and managers, but these are not regular events, and experience elsewhere has indicated that regularity is essential to successful knowledge exchange forums [17].

The TTG will plan and participate in a second "getting research into policy and practice" event in the next twelve months. As effective communication between the two communities should also involve the setting of the research agenda [4] the next forum will focus on identifying a "policy-sensitive" research agenda for future research; the TTG will aim to facilitate taking this forward. This is likely to involve partnering researchers with practitioners and decision-makers to bring a more decision-relevant focus to a research program [1], and to increase the likelihood of research utilisation [20].

A National TTG with a membership comprising falls researchers, policymakers from all State, Territory and Australian Departments of Health, practitioners including allied health practitioners and clinicians, and representatives from non-government sectors such as the aged care industry and the fitness industry would enhance effectiveness. A commitment to meet regularly with the task of working together to identify the research agenda, effect research dissemination, and enhance research uptake into policy and practice in their respective fields and jurisdictions, is essential, and would support the National Plan's goal of knowledge development and dissemination [8]. Additional activities should include identifying and addressing emerging and anticipated constraints, and advocacy to mobilise resources to take forward new programs for preventive health action.

## Conclusion

Researchers and decision-makers see common value in bridging research with policy and practice; there is considerable interest in networking, collaboration and mutual understanding. The forum provided the opportunity for the distillation of research evidence for decision-makers and group discussions highlighted some significant gaps in evidence essential for policymaking. The Falls TTG has the potential to enhance capacity for evidence-informed policy and practice in falls management by providing the infrastructure necessary to actively promote dialogue between stakeholders.

Two activities suggest themselves for the future: the next forum should focus on identifying a "policy-sensitive" research agenda and on partnering researchers and decision-makers in the process; and a National TTG with a broad membership warrants investment.

## Competing interests

SL is a falls researcher with competitive grants for falls research. RP and AZ have no competing interests. The forum received some funding from NSW Health.

## Authors' contributions

All authors contributed to the planning of the forum. SL co-chaired the forum; RP and AZ observed the forum (i.e. undertook data collection). RP analysed the respondent questionnaires and prepared the first draft of the manuscript; AZ and SL contributed to later drafts. All authors read and approved the final manuscript.
